# A 6-year case series of resuscitative thoracotomies performed by a helicopter emergency medical service in a mixed urban and rural area with a comparison of blunt versus penetrating trauma

**DOI:** 10.1186/s13049-022-00997-4

**Published:** 2022-01-26

**Authors:** Phillip Almond, Sarah Morton, Matthew OMeara, Neal Durge

**Affiliations:** Essex and Herts Air Ambulance, Earls Colne, Colchester, Essex CO6 2NS UK

**Keywords:** Resuscitative thoracotomy, Prehospital, Prehospital, Blunt, Penetrating, Traumatic cardiac arrest

## Abstract

**Background:**

Resuscitative thoracotomy (RT) is an intervention that can be performed in the prehospital setting for relieving cardiac tamponade and/or obtaining vascular control of suspected sub-diaphragmatic haemorrhage in patients in traumatic cardiac arrest. The aim of this retrospective case study is to compare the rates of return of spontaneous circulation (ROSC) in RTs performed for both penetrating and blunt trauma over 6 years in a mixed urban and rural environment.

**Methods:**

The electronic records of a single helicopter emergency medical service were reviewed between 1st June 2015 and 31st May 2021 for RTs. Anonymised data including demographics were extracted for relevant cases. Data were analysed with independent t-tests and Χ^2^ tests. A *p* value < 0.05 was considered statistically significant.

**Results:**

Forty-four RTs were preformed within the 6 years (26 for blunt trauma). Eleven ROSCs were achieved (nine blunt, two penetrating) but no patient survived to discharge. In contrast to RTs for penetrating trauma, twelve of the RTs for blunt trauma had a cardiac output present on arrival of the prehospital team (*p* = 0.01). Two patients had an RT performed in a helicopter (one ROSC) and two on a helipad (both achieving ROSC), likely due to the longer transfer times seen in a more rural setting. Four of the RTs for blunt trauma (15%) were found to have a cardiac tamponade versus seven (39%) of the penetrating trauma RTs.

**Conclusion:**

Prehospital RT remains a procedure with low rates of survival but may facilitate a ROSC to allow patients to reach hospital and surgery, particularly when distances to hospitals are greater. A higher-than-expected rate of cardiac tamponade was seen in RTs for blunt trauma, although not caused by a right ventricular wound but instead due to underlying vessel damage.

**Supplementary Information:**

The online version contains supplementary material available at 10.1186/s13049-022-00997-4.

## Background

**Fig. 1:**
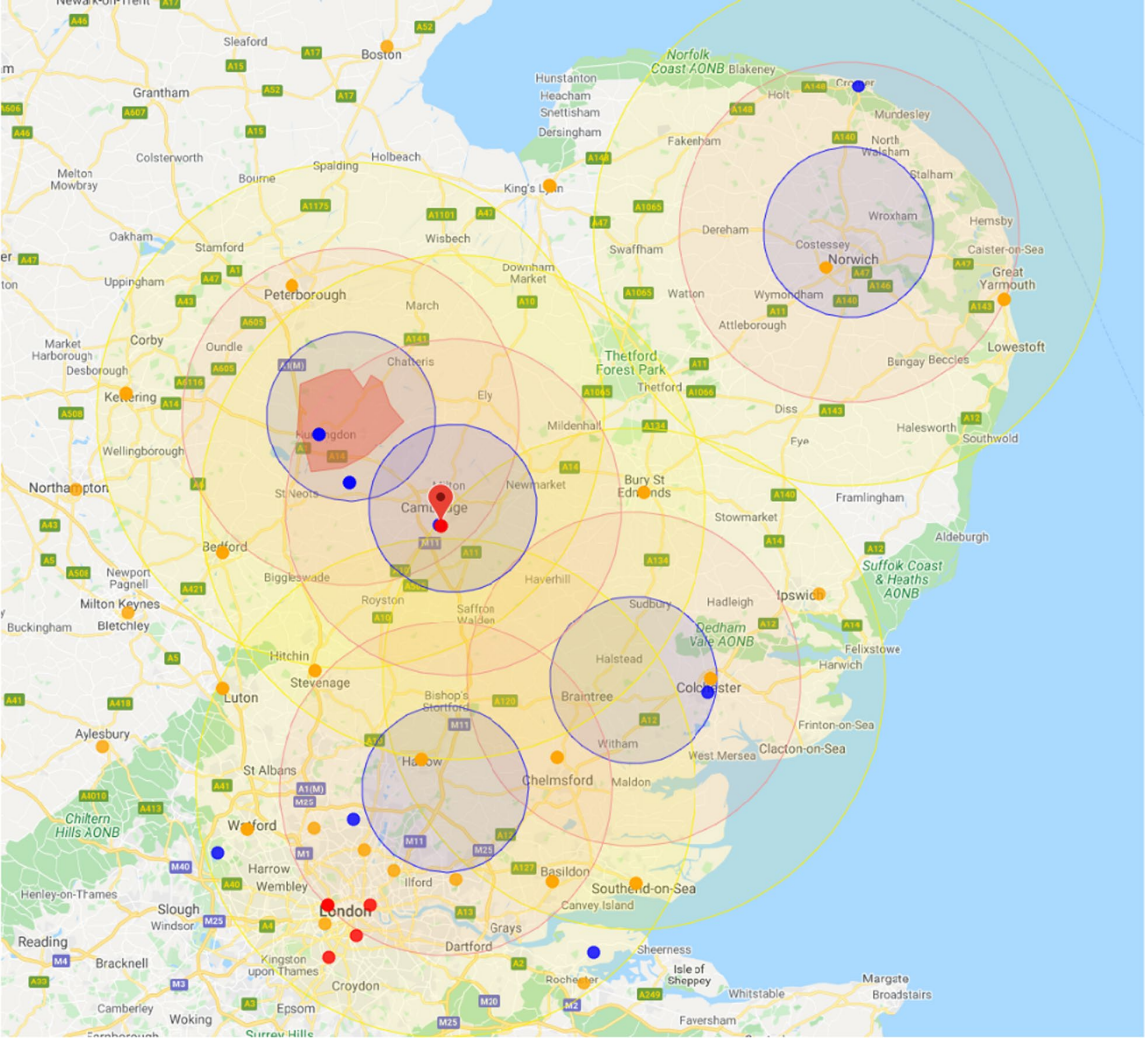
A map of East Anglia showing the location of the five Air Ambulance bases. The concentric circles show tasking radius, small red dots represent Major Trauma Centres, small yellow dots represent Trauma Units (map taken from HEMSBase™)

The East of England Ambulance Service Trust (EEAST) serves a population of approximately 6.2 million people in a mixed urban and rural environment covering an area of approximately 19,114 square kilometres [1]. Within EEAST, three services operate a helicopter emergency medical (HEM) service from five bases around the region, with a physician/paramedic model, including Essex and Herts Air Ambulance Trust (EHAAT) (Fig. 1). EHAAT has operated for a full 24 h since 7th October 2019 (only by car at night). HEMs teams are dispatched when predetermined criteria are satisfied (immediate dispatch), following ‘999’ call interrogation by a critical care paramedic or at the request of the attending medical/paramedical team on-scene. Immediate dispatch criteria includes traumatic cardiac arrest. In addition to serving the East of England population, EHAAT will provide mutual aid for neighbouring ambulance services if requested. EHAAT can provide emergency prehospital Resuscitative Thoracotomy (RT) as an intervention to clinically indicated patients. A Standard Operating Procedure (SOP) exists for this, including indications and timings by which clinicians will operate (see Additional file 2: Appendix 2). All EHAAT clinicians are required to partake in regular training relating to this SOP, including simulation, to ensure maintenance of standards, as it is recognised that, whilst RT is infrequent, clinical skills and decision making must be optimal.

The ability for EHAAT to administer prehospital blood products alongside a RT was introduced in 2019 [2]. The introduction of prehospital blood products has allowed a greater possibility for Trauma Unit (TU) bypass and longer onward transfer to Major Trauma Centre (MTC), where specialist services such as cardiothoracic and vascular surgery is available, as patients can be temporarily stabilised on route to hospital.

In general, RT is considered for patients who have suffered penetrating chest trauma with a view to accessing the chest cavity, relieving a cardiac tamponade, and repair of cardiac injuries [3, 4]. London’s Air Ambulance have shown an 18% survival to hospital discharge following RT for penetrating trauma, with 11 patients out of 13 (85%) having a good neurological outcome [5]. Other studies have struggled to replicate these findings [6]. However, RT can also be used in a select cohort of blunt trauma patients with a view to provide thoracic aortic compression (to allow attempt of haemorrhage control below the diaphragm) and/or repair of clinically presumed organ injury within the chest cavity [3, 4, 7]. Whilst evidence for appropriateness and patient survival from prehospital blunt trauma RT is limited there are an increasing number of publications, incorporating both case studies and case series, showing survivors from the procedure both in the prehospital and hospital emergency department environments [8–12]. One case study reports a neurological intact survivor of a prehospital RT following blunt trauma [11]. In 2016, EHAAT published a case series from April 2010 to April 2016 questioning whether RT for blunt trauma was justified as the evidence and indications remained unclear [13]. However, more recently the European Resuscitation Council has included RT in its 2021 guidelines as an option in traumatic cardiac arrest for both relieving tamponade and obtaining vascular control of sub-diaphragmatic bleeding via manual aortic compression [14].

The aim of this retrospective observational case series is therefore to compare RTs performed for both penetrating and blunt trauma between June 2015 and June 2021 in a mixed urban and rural area to understand more about indications and outcomes.

## Methods

The computerised records (HEMSbase2.0) of a single HEM service (Essex and Herts Air Ambulance Trust) was interrogated to retrospectively extract anonymised data relating to resuscitative thoracotomies performed between 1st June 2015 and 31st May 2021 inclusive. Data relating to case demographics, time of dispatch, aetiology, presence of cardiac output on the arrival of the first emergency medical responders and the HEM service and outcomes were extracted. In addition, information relating to the likely cause of death based on findings at RT or at post-mortem were recorded. Local research policies were consulted; ethical approval was not required [15].

Prehospital administration of blood products became available to the HEM service in 2019 [2]. This consists of three units of Packed Red Blood Cells (PRCs) (reduced to two units from 2020 due to supply issues in the pandemic) and four units of Lyolysed Plasma (LyoPlas™). The use of blood products on scene following its introduction was therefore analysed versus prior to its introduction.

Data was analysed using Microsoft Excel and SPSS statistics (version 26). A Shapiro–Wilk test was performed for normality on continuous values. Mean or median values were calculated as appropriate. Independent t-tests and Χ^2^ tests were calculated to allow comparison between thoracotomies performed for penetrating and blunt trauma. A *p* value < 0.05 was considered statistically significant. The STROBE checklist was followed [16].

## Results

A total of 44 thoracotomies were performed between 1st June 2015 and 31st May 2021, an average of 7.3 per year, including two paediatric thoracotomies and three for patients over the age of 65. 26 were for blunt trauma and 18 were for penetrating. Sadly, there were no survivors to hospital discharge, but 11 patients did achieve return of spontaneous circulation (ROSC) following the thoracotomy. The average time from 999 to HEMS team arrival was 29.9 min. Table 1 shows a summary of the cases attended and Table 2 shows a comparison between the RTs performed for blunt and penetrating trauma. Further details surrounding the cases attended are available in Additional file 1: Appendix 1.

**Table 1:** A summary of cases attended (for more details see Additional file 1: Appendix 1 Table S1)

Case	Type	Age	Gender	Approximate road time to nearest MTC as per Google Maps™ (minutes)	Time from 999 to HEMS arrival (minutes)	Reason Documented for RT	Cardiac output present with any medical provider	Evidence of cardiac movement seen at RT	Cardiac tamponade found at RT	ROSC achieved at any point on scene	Outcome on scene
1	Penetrating	30	Male	45	44	Exclude tamponade	No	No	No	No	PLE
2	Blunt	48	Male	76	20	For aortic compression	No	Yes	No	Yes	GE to trauma unit
3	Blunt	40	Male	57	29	Exclude tamponade/arrest lung haemorrhage + for aortic compression	No	Yes	No	Yes	Aircraft carry to MTC
4	Penetrating	21	Female	62	22	Exclude tamponade	Yes	Yes	No	No	GE to TU
5	Penetrating	20	Male	71	31	Exclude tamponade	Yes	Yes	Yes	No	GE to TU
6	Blunt	49	Male	83	50	For aortic compression	Yes	Not documented	No	No	Aircraft carry to MTC
7	Penetrating	40	Male	50	16	Exclude tamponade	No	No	Yes	No	GE to TU
8	Blunt	40	Male	58	26	For aortic compression	Yes	Not documented	No	No	GE to TU
9	Blunt	62	Male	29	36	For aortic compression	Yes	Yes	No	Yes	Aircraft carry to MTC
10	Penetrating	37	Male	54	26	Exclude tamponade	No	Yes	No	No	PLE
11	Blunt	25	Male	36	14	For aortic compression	Yes	No	No	No	PLE
12	Blunt	50	Female	38	55	For aortic compression	Yes	No	No	No	PLE
13	Penetrating	85	Male	36	29	Exclude tamponade	Yes	No	No	No	PLE
14	Penetrating	45	Female	54	24	Exclude tamponade	No	No	No	No	PLE
15	Penetrating	45	Male	54	24	Exclude tamponade	Yes	Yes	No	No	PLE
16	Penetrating	25	Male	62	31	Exclude tamponade	No	No	No	No	PLE
17	Blunt	50	Male	59	42	Aortic compression	Yes	Yes	No	Yes	GE to TU
18	Blunt	59	Male	47	12	Exclude tamponade/arrest lung haemorrhage + for aortic compression	Yes	Yes	No	Yes	Aircraft carry to MTC
19	Penetrating	35	Male	36	31	Exclude tamponade	No	No	Yes	No	PLE
20	Blunt	58	Female	48	29	Exclude tamponade/ arrest lung haemorrhage + for aortic compression	Yes	Not documented	No	No	PLE
21	Penetrating	25	Male	35	19	Aortic compression	Yes	Yes	No	Yes	GE to TU
22	Blunt	75	Male	27	41	Exclude tamponade/arrest lung haemorrhage	No	Yes	Yes	Yes	GE to MTC
23	Blunt	24	Male	60	35	Exclude tamponade/arrest lung haemorrhage + for aortic compression	Yes	Yes	No	Yes	Aircraft carry to TU
24	Blunt	25	Male	54	40	Exclude tamponade/arrest lung haemorrhage + for aortic compression	Yes	Yes	No	No	PLE
25	Penetrating	17	Male	71	24	Exclude tamponade	Yes	Yes	Yes	Yes	GE to TU
26	Blunt	10	Male	33	15	For aortic compression	No	No	No	No	PLE
27	Penetrating	22	Male	53	15	Exclude tamponade	Yes	No	Yes	No	GE to TU
28	Blunt	47	Male	47	31	Exclude tamponade/arrest lung haemorrhage + for aortic compression	Yes	No	No	No	PLE
29	Blunt	37	Male	65	43	Exclude tamponade/arrest lung haemorrhage + for aortic compression	Yes	Yes	Yes	Yes	GE to TU
30	Penetrating	29	Male	19	25	Exclude tamponade	No	Yes	Yes	No	GE to MTC
31	Penetrating	41	Female	47	31	Exclude tamponade	No	No	No	No	PLE
32	Blunt	66	Male	48	50	Exclude tamponade/arrest lung haemorrhage	Yes	Yes	Yes	Yes	PLE
33	Blunt	47	Male	48	25	Exclude tamponade/arrest lung haemorrhage	Yes	Yes	No	No	PLE
34	Blunt	33	Male	57	29	Exclude tamponade/arrest lung haemorrhage	No	Yes	No	No	PLE
35	Blunt	26	Female	54	29	Exclude tamponade/arrest lung haemorrhage + for aortic compression	Yes	No	No	No	PLE
36	Blunt	59	Male	50	44	Exclude tamponade/arrest lung haemorrhage	No	No	No	No	PLE
37	Penetrating (Gunshot)	59	Male	70	24	Exclude tamponade	Yes	Yes	No	No	PLE
38	Penetrating	37	Male	50	28	Exclude tamponade	No	No	Yes	No	PLE
39	Blunt	25	Female	29	24	Exclude tamponade/arrest lung haemorrhage	Yes	No	No	No	PLE
40	Blunt	55	Male	43	20	For aortic compression	Yes	Yes	No	No	PLE
41	Blunt	30	Male	32	24	Exclude tamponade/arrest lung haemorrhage	No	Yes	Yes	No	PLE
42	Penetrating	34	Male	48	40	Exclude tamponade	No	No	No	No	PLE
43	Blunt	36	Male	63	45	Exclude tamponade/arrest lung haemorrhage	Yes	Yes	No	No	PLE
44	Blunt	26	Male	30	23	Exclude tamponade/arrest lung haemorrhage	Yes	Yes	No	No	PLE

*RT* resuscitative thoracotomy, *PM* post-mortem, *MTC* major trauma centre, *TU* trauma unit, *PLE* pronounced life extinct on scene, *GE* ground escort via ambulance, *ROSC* return of spontaneous circulation

**Table 2:** Summary table comparing RT performed for blunt and penetrating trauma

	RT for blunt trauma	RT for penetrating trauma	
Number performed	26	18	
Male:female	22:4	15:3	*p* = 0.91
Mean age ± standard deviation (years)	42.3 ± 15.9	35.9 ± 16.3	*p* = 0.20
Mean time from 999 to HEMS arrival ± standard deviation (minute)	32.0 ± 11.8	26.9 ± 7.4	*p* = 0.11
Cardiac output present on arrival of first emergency responder			
Yes	18	8	
No	7	10	
Not documented	1	0	*p* = 0.07
Cardiac output present on arrival of HEMS team			
Yes	12	2	
No	14	16	*p = 0.01
Reason for thoracotomy			
To exclude tamponade or arrest lung hemorrhage	8	17	
Aortic control	10	1	
To exclude tamponade and for aortic control	8	0	*p < 0.01
Location of where thoracotomy performed			
Ambulance	1	0	
Helicopter	2	0	
Helipad	2	0	
Emergency department (at request of team)	1	0	
At scene	20	18	*p* = 0.31
Cardiac tamponade present	4/26	7/18	*p* = 0.08
Cardiac movement seen at thoracotomy	15/23 (3 not documented)	8/18	*p* = 0.18
ROSC achieved following thoracotomy	9/26	2/18	*p* = 0.08
Patient outcome from scene			
Aircraft carry to hospital	5	1	
Nearest TU	1	1	
MTC	4	0	
Ground escort to hospital	5	6	
Nearest TU	4	5	
MTC	1	1	
PLE at scene	16	11	*p* = 0.45

*ROSC* return of spontaneous circulation, *TU* trauma unit, *MTC* major trauma centre, *PLE* pronounced life extinct

^*^Statistically significant

Prior to the introduction of prehospital blood products, 12/29 to whom RTs were performed received blood products in a hospital setting of which 6 achieved ROSC. Since the introduction of prehospital blood products in March 2019, EHAAT performed 15 RTs with blood products being administered to 11 of these patients. Of these 11 patients, 5 were penetrating injury and 6 were blunt injury, with no ROSCs achieved. Only one of the eleven patients (9%) was transported to hospital, the remainder were pronounced life extinct on scene versus 13/29 (44.8%) prior to the introduction of blood.

**Fig. 2:**
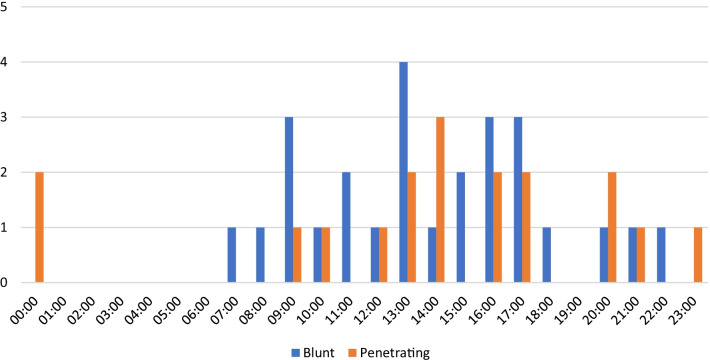
Distribution of timings for initial 999 call for blunt versus penetrating thoracotomies

The distribution of timings for the 999 call is shown below (Fig. 2).

## Discussion

This single UK mixed urban and rural HEM service performed a total of 44 RTs over 6 years, with 58% being for blunt trauma. In total 11 patients achieved ROSC but sadly there were no survivors to hospital discharge. Of note, four out of the 26 RTs for blunt trauma were found to have a cardiac tamponade, of which three achieved at least a brief ROSC, and nine out of the 26 achieved a ROSC.

The lack of survivors to hospital discharge from this cohort of patients differs from the findings of London’s Air Ambulance 20 years ago but is not dissimilar to other services abroad [5, 17]. The survival after an RT in the emergency department is estimated at 7.4%; this dropped to 1.4% for blunt RTs [18]. Our findings may therefore be influenced by the higher percentage of RTs for blunt trauma and longer run times to incidents and MTCs. Table One (and Table S1 Additional file 1: Appendix 1) details the extensive distance and travel times (by road only) to the nearest MTC for many of the patients, with a mean distance of 47.5 km (range 3.7–105.6 km) and mean time of 49.7 min (range 19–83 min). Whilst it is possible to transport a patient to whom an RT has been performed in an aircraft and thus reduce the transport time to an MTC, there would be significant difficulties in loading and unloading of these patients and a high possibility of deleterious effects to patient management and care. This is further complicated by the fact that several of the MTC helipads that EHAAT utilise when flying a patient require a secondary transfer by ambulance. For London’s Air Ambulance, the mean time to scene in a RT group was 9.29 min versus in this study at least 29.9 min from 999 to arrival on scene of the HEM service [19]. It has been suggested that RTs need to be performed within 30 min of the injury to have a better chance of survival, and these distances and times make this challenging despite rapid dispatch of the HEM service [20]. There does however appear to be a learning curve and it may be that if numbers increase that long term survivors may be seen [21]. It is also encouraging that, despite the distances to hospital and longer run times to incidents, patients are gaining a ROSC and therefore access to surgery to give them the chance of survival, suggesting that it is worth performing.

When looking at the indications for RT, in the blunt cohort there was a statistically significant increased percentage who had cardiac output (i.e. a central pulse) on arrival of the HEM service (46%) who then went on to require a RT versus penetrating trauma where the vast majority (89%) did not have a cardiac output on arrival of the HEM service. This fits with the suggestion in the literature that an RT should only be performed in patients with blunt thoracic trauma if they have received less than 5 min of CPR, as anything beyond this is considered futile [3, 22]. For in-hospital RTs performed for abdominal exsanguination a review suggests a 0–16% survival rate, which may be slightly higher if it is an isolated iliac injury [9]. However, blunt trauma does appear to have worse outcomes than penetrating [9]. For a prehospital London cohort, 25 patients (47.1%) had no signs of life on arrival of the team, with only one of these 25 patients surviving [19]. Performing a RT when there are signs of life on arrival (for example palpable pulses or respiratory effort) of the first crew is likely to be more successful (9/10 survivors in the London cohort had signs of life on arrival of the crew), although the London data is skewed towards penetrating trauma [19]. The findings of this study therefore appears to support that a blunt RT should only be performed on patients with signs of life on the first emergency responder arrival, or perhaps even the HEMS team arrival, with then witnessed loss of central pulses, as these patients are most likely to be the survivors [19]. Currently, ultrasound to look for cardiac tamponade is not part of the SOP for RT in our service and this too may be something that should be included in the future.

Whilst the vast majority of both penetrating and blunt RTs occurred on scene, two were performed in a helicopter and two on a helipad. ROSC was achieved in three of them. To our knowledge this has not been previously described in the literature. It is important to note that there were no unexpected complications and no needlestick or similar injuries for clinical staff in any of the RTs, including the ones performed in the helicopter or on a helipad. This suggests that whilst this may not be ideal, it is possible to perform RTs in this setting. One study has shown that rapid sequence induction can be performed in a helicopter cabin [23]. In environments where distances to hospital deem aircraft carries necessary, training for in-flight thoracotomies could be considered. The newer airframes being utilised by air ambulances in the UK, with larger cabin space and 360° access to the patient, would potentially make this procedure viable; however, this requires further research and cannot yet be recommended.

It is interesting to note that, in one of the penetrating patients the indication for RT was for aortic control and not to exclude tamponade as the injury was to the femoral artery (see case 21, Table 1, Additional file 1: Appendix 1). In contrast 69% of thoracotomies performed for blunt trauma were performed with an aim of achieving some element of aortic control for sub-diaphragmatic haemorrhage. This suggests that there may be value in exploring the benefits of endovascular resuscitation techniques, such as REBOA in a mixed urban and rural setting. Despite clinicians initially intending to perform the RT for aortic control of sub-diaphragmatic haemorrhage, 15% (4/26) of the blunt RTs were shown to have a cardiac tamponade, higher than may be expected in blunt trauma patients. However, unlike case reports of survivors of blunt cardiac tamponade normally secondary to right ventricular injuries, none of the cases reported here were found to have a right ventricular injury [24]. Three patients were found to have vessel damage (either the left anterior descending coronary artery or the aortic root) and for one patient it was unclear but no ventricular damage could be found. These underlying mechanisms of the tamponade are likely to be much more technically difficult to repair than a single ventricular wound, but relief of the tamponade may facilitate a timely transfer to hospital for further surgery, as was shown for several of the patients in this case series.

Overall, the injuries for both groups identified at RT or post-mortem show similar patterns to previous studies [25]. Survivors do tend to be seen in patients with a single right ventricle wound rather than more complex injury patterns, another reason for the lack of survivors seen in this case series [5, 25]. In addition, there are some patients described in this cohort that had additional injuries, particularly head injuries; it can be difficult on scene to determine the exact cause of cardiac arrest and an RT is performed as an intervention to exclude a “potentially treatable” cause of cardiac arrest, such as a cardiac injury. Other prehospital services can offer techniques such as REBOA for abdominal or pelvic haemorrhage and it may be that, as this becomes more widespread, this current indication for thoracotomies, particularly in blunt trauma, decreases and survival rate increases [26]. However, as studies have shown, RT is still valid for attempting to slow or cease abdominal exsanguination through aortic compression [9].

The mean age of the patients seen in this study were 42.3 years for RT in blunt trauma and 35.9 years for RT in penetrating trauma, with three patients having a RT aged over 65. This is a similar average age seen in other studies [19, 22, 25]. It is recognised that an age over 60 is an independent predictor of mortality [19, 22]. The gender distribution also remains consistent with other studies [22].

Following the prehospital administration of blood products, fewer patients were transported to hospital, with the majority pronounced life extinct on scene. This may be because now all the most significant interventions can be offered on scene without the need to transfer the patient. Of the 6 RTs performed for blunt trauma, the primary reasons for RT as detailed in the notes were: chest injury (4/6), aortic control (of sub-diaphragmatic haemorrhage) (1/6) and chest injury and aortic control (of sub-diaphragmatic haemorrhage) (1/6). The data appears to confirm that the introduction of blood products to the service did not encourage excessive RTs in blunt trauma patients and that the primary reason for performing RT was based on clinical assessment of a potentially treatable internal chest injury with no change in the average number of RTs per year.

### Limitations

This study is a single centre cohort study. However, it does show a cohort based in a rural and urban environment, a different environment to many previous studies that are purely urban in nature. In the future, it would be interesting to combine findings with other HEM services operating in a similar setting. The likely cause of death is based on a combination of post-mortem results and findings at RT so may not be fully accurate. However, without post-mortem reports on every patient it is unlikely that a better insight than this will be gained. This may be an area to explore for the service in the future as improved post-mortem reporting and recording will allow a comparison of presumed versus actual injury loads to validate if the service is selecting the correct subset of patients for RT.

## Conclusion

Prehospital RTs remains a procedure with low rates of survival but in areas where there are longer transfer times to hospitals patients are given the optimal chance of survival by the procedure being performed prehospital. As such, no difference in survival nor ROSC was seen between RTs performed for blunt and penetrating trauma in this case series. Cardiac tamponade can occur in blunt trauma patients, but this study shows that the cause is less likely to be a right ventricular wound but instead be due to underlying vessel damage.

## Supplementary Information

**Additional file 1.** **Table S1:** A Detailed Description of Cases Attended.

**Additional file 2.** EHAAT's Pre-hospital Standard Operating Procedure Resuscitative Thoracotomy: March 2021.

## Data Availability

All data generated or analysed during this study are included in this published article.
