# Supplementary material for: A 6-year case series of resuscitative thoracotomies performed by a helicopter emergency medical service in a mixed urban and rural area with a comparison of blunt versus penetrating trauma
Source: Scand J Trauma Resusc Emerg Med. 2022 Jan 26;30:8. doi: 10.1186/s13049-022-00997-4 (PMC8793242; doi:10.1186/s13049-022-00997-4)
Supplement: Supplementary file 1 — Additional file 1. Table S1: A Detailed Description of Cases Attended. [file 13049_2022_997_MOESM1_ESM.docx]

*Appendix 1 Table 1: A Detailed Description of Cases Attended*

| **Case** | **Type** | **Age** | **Gender** | **Approximate road distance & time to nearest MTC as per Google Maps™** | **Time from 999 to first EMS crew on scene (minutes)** | **Time from 999 to dispatch of HEMS crew (minutes)** | **Time from 999 to HEMS arrival (minutes)** | **Reason Documented for RT** | **Cardiac output present on first EMS arrival** | **Cardiac output present on HEMS crew arrival** | **Location of RT** | **Blood products administered (Prehospital/hospital)** | **Evidence of cardiac movement seen at RT** | **Cardiac tamponade found at RT** | **ROSC achieved at any point on scene** | **Outcome on scene** | **Outcome in hospital** | **Injuries found at RT/PM/ hospital** |
| --- | --- | --- | --- | --- | --- | --- | --- | --- | --- | --- | --- | --- | --- | --- | --- | --- | --- | --- |
| 1 | Penetrating | 30 | Male | 39.7km 45 minutes | 34 | 3 | 44 | Exclude tamponade | No | No | Scene | None | No | No | No | PLE | - | LV outflow tract wound |
| 2 | Blunt | 48 | Male | 73.1km  76 minutes | 11 | 3 | 20 | For aortic compression | Not documented | No | Scene | 1000ml saline only | Yes | No | Yes | GE to trauma unit | Trauma unit attended – FAST negative, bilateral acetabular fractures, right sided superior and inferior rami fractures. Transferred to MTC with aortic pressure release and blood products running. At MTC, theatre for laparotomy and pelvic packing, PLE within 24 hours. | Nil significant – PM recorded hypoxic cause of arrest |
| 3 | Blunt | 40 | Male | 47.3km  57 minutes | 3 | 10 | 29 | Exclude tamponade/arrest lung haemorrhage + for aortic compression | No | No | In flight to MTC | 1000ml saline and 1 x PRC on pad | Yes | No | Yes | Aircraft carry to MTC | Hospital PLE (no further information available) | Head and chest injuries |
| 4 | Penetrating | 21 | Female | 49.2km  62 minutes | 5 | 4 | 22 | Exclude tamponade | Yes | No | Scene | Saline only (volume not documented). Blood given in ED (volume unclear) | Yes | No | No | GE to TU | TU PLE. Massive pulmonary haemorrhage enroute to hospital; lung twist performed. | Lung wound |
| 5 | Penetrating | 20 | Male | 67.8km  71 minutes | 13 | 7 | 31 | Exclude tamponade | Yes | No | Scene | Saline (volume not documented) | Yes | Yes | No | GE to TU | Hospital PLE (TU) Patient went to theatre but no other information on hospital interventions | RV wound |
| 6 | Blunt | 49 | Male | 124.4km  83 minutes | 13 | 25 | 50 | For aortic compression | Yes | Yes | In flight to MTC | 1500 ml saline, 2 x PRC on pad | Not documented | No | No | Aircraft carry to MTC | Hospital PLE - ROSC in ED following blood, no further info on hospital interventions. | Hypovolaemia secondary to pelvic fracture |
| 7 | Penetrating | 40 | Male | 43.6km  50 minutes | 4 | 8 | 16 | Exclude tamponade | No | No | Scene | 1000ml saline | No | Yes | No | GE to TU | Hospital PLE (TU) Surgeons not available as in theatre, immediate blood transfusion, vascular surgeon tasked from MTC to TU. Found to have irreparable through and through injury of heart. | LV wound |
| 8 | Blunt | 40 | Male | 38.9km  58 minutes | 17 | 9 | 26 | For aortic compression | Yes | Yes | In trauma unit by prehospital team | 3500ml saline | Not documented | No | No | GE to TU | Hospital PLE (TU) General surgeon present but HEMS performed thoracotomy in ED, no tamponade, aortic compression continued as patient taken to theatre, retroperitoneal haematoma with significant injury burden, PLE in theatre. | Hypovolaemia secondary to retroperitoneal haematoma |
| 9 | Blunt | 62 | Male | 23.7km  29 minutes | 8 | 12 | 36 | For aortic compression | Yes | Yes | MTC helipad | 750 ml saline, 2 x PRC on pad | Yes | No | Yes | Aircraft carry to MTC | Hospital PLE (MTC) Strong ROSC following blood in ED, taken to theatres, splenectomy, ongoing cardiac arrests when attempting to close – PLE in theatre | Hypovolaemia secondary to pelvic fracture and abdominal organ injuries. |
| 10 | Penetrating | 37 | Male | 48.9km  54 minutes | 5 | 4 | 26 | Exclude tamponade | No | No | Scene | 1500ml saline | Yes | No | No | PLE | - | Lung wound |
| 11 | Blunt | 25 | Male | 25.9  36 minutes | 1 | 4 | 14 | For aortic compression | Yes | No | Scene | 1000ml saline | No | No | No | PLE | - | Lung wound |
| 12 | Blunt | 50 | Female | 37.5km  38 minutes | 8 | 23 | 55 | For aortic compression | Yes | Yes | Scene | 500ml saline | No | No | No | PLE | - | Hypovolaemia secondary to pelvic fracture |
| 13 | Penetrating | 85 | Male | 38.0km  36 minutes | Not documented | 7 | 29 | Exclude tamponade | Yes | No | Scene | None | No | No | No | PLE | - | RV wound |
| 14 | Penetrating | 45 | Female | 47.5km  54 minutes | 8 | 4 | 24 | Exclude tamponade | No | No | Scene | 1000ml saline | No | No | No | PLE | - | Lung wound |
| 15 | Penetrating | 45 | Male | 47.5km  54 minutes | 8 | 4 | 24 | Exclude tamponade | Yes | Yes | Scene | 2000ml saline by first responders | Yes | No | No | PLE | - | Hypovolaemia secondary to neck wound |
| 16 | Penetrating | 25 | Male | 69.0km  62 minutes | 10 | 1 | 31 | Exclude tamponade | No | No | Scene | 500ml saline | No | No | No | PLE | - | Lung wound |
| 17 | Blunt | 50 | Male | 84.0km  59 minutes | 16 | 22 | 42 | Aortic compression | Yes | Yes | Scene | Saline (volume not documented) Blood given in TU | Yes | No | Yes | GE to TU | Hospital PLE (TU). Blood products in TU, episode of VF, manual internal cardiac massage and external defibrillation, decision to cease resus | Head injury and hypovolaemia secondary to pelvic fracture |
| 18 | Blunt | 59 | Male | 52.1km  47 minutes | 9 | 5 | 12 | Exclude tamponade/arrest lung haemorrhage + for aortic compression | Yes | Yes | MTC helipad | 2 x units PRC (on helipad) | Yes | No | Yes | Aircraft carry to MTC | Hospital PLE (MTC) Blood transfusion continued in MTC for 45 mins, internal defibrillation but no ROSC | Lung wound |
| 19 | Penetrating | 35 | Male | 32.2km  36 minutes | 7 | 6 | 31 | Exclude tamponade | No | No | Scene | 3000ml saline | No | Yes | No | PLE | - | RV wound |
| 20 | Blunt | 58 | Female | 41.7km  48 minutes | 9 | 13 | 29 | Exclude tamponade/ arrest lung haemorrhage + for aortic compression | Yes | Yes | Scene | 3000ml saline, 300ml hypertonic | Not documented | No | No | PLE | - | Intracranial injuries, contusions, intrathoracic bleeds from intercostal arteries, bilateral flail segments, liver laceration, femoral#, humeral#, soft tissue bleeds throughout |
| 21 | Penetrating | 25 | Male | 28.8km  35 minutes | 11 | 4 | 19 | Aortic compression | Yes | No | Scene | None | Yes | No | Yes | GE to TU | Hospital PLE (TU) 4 units of blood but no ROSC so decision to cease resus in TU. | Hypovolaemia secondary to femoral artery wound |
| 22 | Blunt | 75 | Male | 26.2km  27 minutes | 7 | 27 | 41 | Exclude tamponade/arrest lung haemorrhage | No | Yes | In ambulance | None | Yes | Yes | Yes | GE to MTC | Hospital PLE (MTC) On scene - left tension pneumothorax and serous blood, right side haemothorax, ~400ml cardiac tamponade relieved but bleeding point not identified. ROSC achieved. Repeat cardiac arrest on arrival at MTC, deemed nonsurvivable injuries (head and chest) PLE. | Left anterior descending graft wound plus head injuries |
| 23 | Blunt | 24 | Male | 57.6km  60 minutes | 7 | 6 | 35 | Exclude tamponade/arrest lung haemorrhage + for aortic compression | Yes | Yes | Scene | 1000ml saline Blood in TU | Yes | No | Yes | Aircraft carry to TU | Hospital PLE (TU) – ROSC enroute to TU, lung laceration on right lower lobe so hilum clamped, ongoing internal cardiac massage support required enroute. | Hypovolaemia secondary to abdominal organ injury |
| 24 | Blunt | 25 | Male | 48.4km  54 minutes | 6 | 29 | 40 | Exclude tamponade/arrest lung haemorrhage + for aortic compression | Yes | Yes | Scene | EEAST 200ml 10% Dextrose, EHAAT 2000ml saline, 500ml hypertonic | Yes | No | No | PLE | - | Hypovolaemia secondary to pelvic fracture |
| 25 | Penetrating | 17 | Male | 105.6km  71 minutes | Not documented | 4 | 24 | Exclude tamponade | Yes | No | Scene | 1000ml saline | Yes | Yes | Yes | GE to TU | Hospital PLE (TU). 60 mins of ongoing resuscitation, surgical team re-explored chest cavity, patient deemed too unstable to transfer (no further information available.) | LV wound and left lung wound |
| 26 | Blunt | 10 | Male | 26.7km  33 minutes | 9 | 3 | 15 | For aortic compression | No | No | Scene | 1000ml saline | No | No | No | PLE | - | Hypovolaemia secondary to pelvic fracture |
| 27 | Penetrating | 22 | Male | 51.3km  53 minutes | 3 | 3 | 15 | Exclude tamponade | Yes | No | Scene | None  3 x PRC at TU | No | Yes | No | GE to TU | Hospital PLE (TU) Vascular surgeon clamed aorta, 3 x PRC to no improvement, decision to cease resus following 30 mins of cardiac arrest | Proximal aortic injury |
| 28 | Blunt | 47 | Male | 28.5km  47 minutes | 9 | 21 | 31 | Exclude tamponade/arrest lung haemorrhage + for aortic compression | Yes | No | Scene | None (other patient on scene required blood and was deemed to have better survival chance) | No | No | No | PLE | - | Hypovolaemia secondary to abdominal organ injury |
| 29 | Blunt | 37 | Male | 51.0km  65 minutes | 17 | 6 | 43 | Exclude tamponade/arrest lung haemorrhage + for aortic compression | Yes | No | Scene | EEAST 500ml saline, EHAAT 500ml saline, 3 x PRC | Yes | Yes | Yes | GE to TU | Hospital PLE (TU) Blood via right atrial catheter, aortic compression continued and internal massage, mild fibrillation seen, adrenaline given to little effect, discussion with MTC but no change so PLE 30 mins after arrival at TU. | Cardiac tamponade and hypovolaemia secondary to abdominal organ injury |
| 30 | Penetrating | 29 | Male | 3.7km  19 minutes | Not documented | Not documented | 25 | Exclude tamponade | No | No | Scene | 2 x PRC, 1 x LyoPlas™, 500ml saline | Yes | Yes | No | GE to MTC | Hospital PLE (MTC) theatre following transfusion and ROSC, ABG in theatre revealed worsening acidosis (pH 6.5) intra-abdominal blood found in theatre with unclear source, surgery abandoned due to futility. | Cardiac tamponade and hypovolaemia secondary to abdominal organ injury – |
| 31 | Penetrating | 41 | Female | 44.1km  47 minutes | 12 | 16 | 31 | Exclude tamponade | No | No | Scene | None | No | No | No | PLE | - | Nil obvious injuries - tension pneumothorax suspected on PM |
| 32 | Blunt | 66 | Male | 35.9km  48 minutes | 8 | 5 | 50 | Exclude tamponade/arrest lung haemorrhage | Yes | No | Scene | None | Yes | Yes | Yes | PLE | - | Lung wound |
| 33 | Blunt | 47 | Male | 24.3km  48 minutes | 7 | 10 | 25 | Exclude tamponade/arrest lung haemorrhage | Yes | No | Scene | None | Yes | No | No | PLE | - | Lung wound |
| 34 | Blunt | 33 | Male | 65.0km  57 minutes | 14 | 7 | 29 | Exclude tamponade/arrest lung haemorrhage | No | No | Scene | 2x PRC, 2 x LyoPlas™ | Yes | No | No | PLE | - | Lung wound |
| 35 | Blunt | 26 | Female | 42.8km  54 minutes | 7 | 4 | 29 | Exclude tamponade/arrest lung haemorrhage + for aortic compression | Yes | Yes | Scene | 2 x PRC, 2 x LyoPlas™ | No | No | No | PLE | - | PM report of tracheal injury suggestive of impalement, which penetrated thyroid cartilage, trachea, and vasculature |
| 36 | Blunt | 59 | Male | 33.2km  50 minutes | 9 | 7 | 44 | Exclude tamponade/arrest lung haemorrhage | No | No | Scene | EEAST 1000ml saline, EHAAT 1 x unit PRC | No | No | No | PLE | - | Lung wound |
| 37 | Penetrating (Gunshot) | 59 | Male | 92.4km  70 minutes | 3 | 1 | 24 | Exclude tamponade | Yes | Yes | Scene | 2 x PRC, 1 x LyoPlas™ | Yes | No | No | PLE | - | Lung wound |
| 38 | Penetrating | 37 | Male | 34.3km  50 minutes | Not documented | Not documented | 28 | Exclude tamponade | No | No | Scene | 1 x unit PRC | No | Yes | No | PLE | - | Cardiac tamponade |
| 39 | Blunt | 25 | Female | 28.2km  29 minutes | 19 | 2 | 24 | Exclude tamponade/arrest lung haemorrhage | Yes | No | Scene | 1 x unit PRC | No | No | No | PLE | - | Traumatic brain injury |
| 40 | Blunt | 55 | Male | 40.6km  43 minutes | 9 | 2 | 20 | For aortic compression | Yes | No | Scene | 1 x unit PRC | Yes | No | No | PLE | - | Liver wound and hypovolaemia |
| 41 | Blunt | 30 | Male | 28.3km  32 minutes | 4 | 3 | 24 | Exclude tamponade/arrest lung haemorrhage | No | No | Scene | EEAST 1200ml saline, EHAAT 2 x PRC, 1 x LyoPlas™ | Yes | Yes | No | PLE | - | Cardiac tamponade and aortic arch rupture |
| 42 | Penetrating | 34 | Male | 47.6km  48 minutes | 9 | 15 | 40 | Exclude tamponade | No | No | Scene | 1 unit PRC | No | No | No | PLE | - | Hypovolaemia secondary to abdominal organ wounds |
| 43 | Blunt | 36 | Male | 98.5km  63 minutes | 5 | 13 | 45 | Exclude tamponade/arrest lung haemorrhage | Yes | Yes | Scene | 2 units PRC | Yes | No | No | PLE | - | Hypovolaemia secondary to abdominal organ injury |
| 44 | Blunt | 26 | Male | 13.7km  30 minutes |  | 2 | 23 | Exclude tamponade/arrest lung haemorrhage | Yes | No | scene | LAS 500ml saline, EHAAT 1 unit PRC | Yes | No | No | PLE | - | Hypovolaemia from abdominal organ injury, Neck injury haematoma |

**RT=Resuscitative Thoracotomy; PM = post-mortem; MTC = major trauma centre; TU = trauma unit; PLE = pronounced life extinct on scene; GE = ground escort via ambulance; LV = left ventricle; RV = right ventricle; PRC = packed red cell; ETCO_2_ = end tidal carbon dioxide; ROSC = return of spontaneous circulation; VF = ventricular fibrillation; ABG = arterial blood gas*
