# Supplementary material for: A 6-year case series of resuscitative thoracotomies performed by a helicopter emergency medical service in a mixed urban and rural area with a comparison of blunt versus penetrating trauma
Source: Scand J Trauma Resusc Emerg Med. 2022 Jan 26;30:8. doi: 10.1186/s13049-022-00997-4 (PMC8793242; doi:10.1186/s13049-022-00997-4)
Supplement: Supplementary file 2 — Additional file 2. EHAAT's Pre-hospital Standard Operating Procedure Resuscitative Thoracotomy: March 2021. [file 13049_2022_997_MOESM2_ESM.pdf]

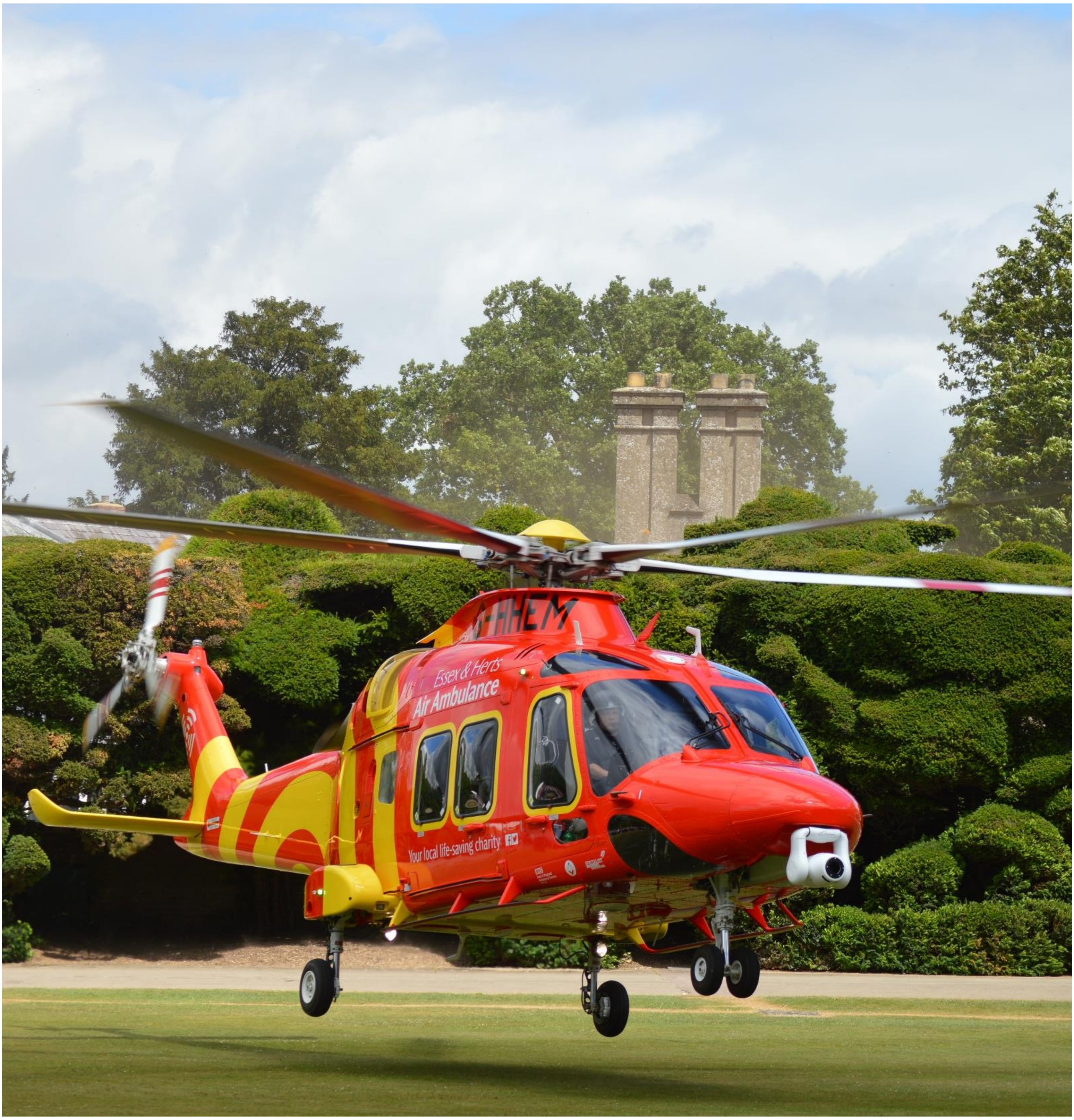

Pre-hospital Care Standard Operating Procedure Resuscitative Thoracotomy

March 2021

Essex & Herts
Air Ambulance
Your local life-saving charity

CONTENTS

1.	Aims	3
2.	Background	3
3.	Indications	3
4.	Contents of Thoracotomy Kit	4
5.	Procedure	4
6.	Triage and Disposition	7
7.	Documentation and Audit	7
8.	References	8

1.0 Aims

- To ensure that all staff are familiar with the indications, process and procedure for delivering resuscitative thoracotomy in the pre-hospital setting.
- To define the patient population that the process may benefit.
- To describe indications for resuscitative thoracotomy
- To describe the contents of the thoracotomy set.
- To describe the operative process.

2.0 Background

Patients who lose their vital signs following trauma have a poor prognosis. Transportation to hospital whilst undertaking cardiopulmonary resuscitation is rarely of value; blood flow through the heart may be obstructed or the heart may be empty through hypovolaemia.

Resuscitative thoracotomy (RT) for penetrating chest injury is primarily to address one specific group of patients; those with a *simple cardiac wound* leading to *tamponade* and cardiac arrest. To maximise the chance of survival, surgical intervention should be immediate (within 10 minutes of loss of vital signs).

As the majority of tamponades are clotted, pericardiocentesis is unlikely to succeed and formal thoracotomy and pericardiotomy is necessary. Patients may also require internal cardiac massage to gain return of spontaneous cardiac activity.

RT in blunt trauma has a very poor prognosis. There is a move in-hospital to limit this to patients who are suspected to have arrested due to an underlying chest injury. For example multiple left sided rib fractures with suspected penetrating cardiac injury/tamponade, or sudden anterior/posterior deceleration injury to the chest with risk of IVC tear or atrial appendage blow out. In the pre-hospital setting patients that have a severe head injury, or are thought to have exsanguinated primarily from a massive injury burden below the diaphragm should not undergo RT.

In the ‘peri-arrest’ patient, the decision on whether or not to perform a prehospital thoracotomy vs rapid transfer to hospital will depend largely on the distribution of wounds and what the clinician ‘expects to find’ (i.e. a readily treatable disease such as tamponade vs an aortic injury or high energy lung laceration from gunshot wound).

3.0 Indications

These indications are a guide; they are not absolutes and do not cover every eventuality.

- A patient in cardiac arrest or in extremis, with penetrating injury to the chest, abdomen, neck, axilla or groin.
- Penetrating limb injury causing cardiac arrest, where immediate control of bleeding and vascular access cannot be achieved.
- Cardiac arrest following blunt trauma, where there were recent signs of life, absence of catastrophic head injury (based on history or clinical examination) and where it is felt that a treatable chest injury is the primary cause of arrest.

4.0 Contents of the Thoracotomy Kit

- **Autoclaved items:**
- Finochietto Rib Spreader X1
- Curved Mayo Scissors X1
- Spencer Wells Forceps X2
- Mosquito Artery Forceps X2
- Satinsky Clamp X1

Non-autoclaved items are found in the base compartment and open section of the chest surgery module:

- Chloraprep with tint X2
- Size 22 retractable scalpel X2
- Gigli Saw Wire and Handles (pair) X2
- Spencer Wells Forceps X2
- Tuffcut Shears X1
- Silk suture on a curved hand needle X2
- Foley Catheter X1
- Face Mask with Visor X4
- Staple gun X1
- Long sleeve gloves S/M/L X2
- Thoracotomy set checklist

5.0 Procedure

5.1 Process

- Once traumatic cardiac arrest is confirmed and thoracotomy is indicated, the decision to undertake a thoracotomy should be made within 10–15 seconds. This is a clinical diagnosis and should not require monitoring.

- The patient should be rapidly moved to an area where there is 360 degrees of access to perform the procedure.

- Appropriate Personal Protective Equipment should be worn, including sterile gloves. Good lighting/head torches should be strongly considered.

- The primary focus of the medical team is to perform the rapid RT. Other resources can be directed to perform intubation or IV access simultaneously if available.

- Identify the markings for the 4th intercostal space. The 4th intercostal space at the mid-axillary line lies approximately level with the nipple in men and the inframammary fold in women.

- The doctor and paramedic should undertake bilateral simple thoracostomies using a size 22 scalpel blade and Spencer Wells forceps making a note of any wounds already in situ for forensic reasons.

- Make a skin incision along the line of the 4th interspace, joining both thoracostomies in an upside-down swallow shape under both areolae and up towards the sternum. The incision should aim to get through all skin layers and fat down to the intercostal

muscles. For safety this is performed by a single operator.

- Using a pair of scissors extend the thoracostomies on both sides up to the breastbone in the line of the 4th space. If the two sides of the thoracotomy are in different rib spaces do not cut through ribs; instead cut the sternum at an angle.

- It is often possible to cut through the sternum with the Tuffcut shears. If not, the sternum should be divided with a Gigli saw. Pass Spencer Wells forceps behind the sternum, grasp the Gigli wire and pull it behind the sternum. Attach the wire to the Gigli handles and saw. It should take little more than 2 or 3 pulls. Caution should be taken to avoid splashes and injury to clinicians as the Gigli wire passes through the sternum under tension.

- Extend the incision in the intercostal space posteriorly to the posterior axillary line.

- Open the chest wide and use suction if necessary to help clear the field and identify anatomy.

- The Finochietto rib spreaders can be inserted at this stage to obtain maximal exposure.

- Rub the connective tissue away to expose the pericardium.

- If tamponade is present the pericardium may look tense. Open the pericardium even if there is no visible tamponade to inspect the heart and perform massage. Raise a “tent” of pericardium anteriorly, then cut a small vertical hole. Extend the hole fully upwards to the aorta and downwards vertically with curved scissors. Be careful not to cut the atrial appendages trying to escape the pericardium. The cut can be extended to become an inverted ‘T’ to assist in the full ‘delivery’ of the heart. Posterior wounds may be masked if the heart is not fully delivered.

- Remove any blood clots using large swabs and with your hands. The heart may fibrillate or beat spontaneously. Observe where blood is coming from and control as below.

- If the heart makes no spontaneous movement, try flicking it with your finger. If no movement comes about, begin internal cardiac massage. Focus on the quality of massage you are providing. Use a two-handed technique and ensure the heart is flat in its bed and not kinked across its axis.

- Get an assistant to compress the descending aorta against the spinal column; this may require careful coaching in the inexperienced. The descending thoracic aorta sits anterolateral to the vertebral body. Aortic compression over a prolonged period is harmful, at an appropriate stage consider the risk vs. benefit of releasing aortic compression.

- By this time IV access should have been established. Load the heart with volume - you will feel whether it is empty or not. The right atrium may be considered for access if absolutely necessary.

- If there is any evidence of developing spontaneous cardiac motion, do not immediately stop internal cardiac massage. Massage should be continued until myocardial activity is good and sustained.

- If the procedure is successful the internal thoracic arteries might bleed and require ligation/clipping. Look for them and clip/tie them off.

- Spontaneous ventilation may occur once pulsatile flow is established.

- Anaesthetise the patient as required.

n.b It is envisaged that many of the above activities would take place concurrently.

5.2 Cardiac Wounds

- Bleeding from a cardiac wound should be controlled directly with a finger (or two) gently applied to the area being careful not to extend the wound. A silk suture on a hand needle or staples can be used to close the wound. Foley catheters may also have a role in temporarily occluding wounds but should be used with caution as they risk extending the wound and reducing stroke volume.

- Small wounds can be closed with staples. Caution is advised if used to close wounds involving the right ventricle due to the thin ventricular wall

- Wounds adjacent to coronary arteries should be closed avoiding the artery using a horizontal mattress suture. If the artery is very distal, a simple suture may be placed, accepting that blood flow to the distal myocardium will be lost.

5.3 Lung Injury

- Consider using a Satinsky Clamp, blast bandage and direct compression to arrest any bleeding.

- To arrest haemorrhage a hilar twist may be considered after freeing the inferior pulmonary ligament. The lower lobe is rotated anteriorly over the upper lobe. Freeing the inferior pulmonary ligament will also allow better access to the aorta.

5.4 Manual Aortic Occlusion

- If it appears that exsanguination is due to a non-cardiac cause, compression of the descending aorta may arrest bleeding and maximise coronary and cerebral perfusion.
- The descending thoracic aorta typically lies anterolateral to the vertebral column. The aorta can sometimes be more lateral, depending on how high above the diaphragm compression is placed.
- Entering the left hemithorax, proceed behind the left lung until the spine is felt. Using fingers compress the soft tissue structures against the spine’s anterolateral surface. Do not attempt to clamp the aorta.
- If the heart is adequately filled, a pulse is often felt from spontaneous or augmented cardiac compression.

5.5 Ventricular Fibrillation

- In the young patient with a structurally normal heart, ventricular fibrillation is most commonly associated with inadequate coronary blood flow. In fine VF the priority is to continue good quality internal cardiac massage and optimise filling.
- Defibrillation is appropriate in coarse VF. It is possible to defibrillate with the chest open, as modern biphasic defibrillators will compensate for increased impedance. If

this is unsuccessful, closing the chest prior to defibrillation should be considered.
- Ensure that no one is touching the patient and that all hands are out of the chest before defibrillating.

5.6 Common Reasons for Failure

- Delay in opening the chest
- Anterior location of thoracostomies preventing adequate access.
- Failure to open the pericardium and identify wounds
- Single-handed, poor quality, intermittent massage
- Failure to occlude aorta
- Kinking of heart anteriorly, delivering it through inadequate pericardial incision, impairing vascular filling.

5.7 Key Points for Success

- Rapid access - < 1 minute to pericardial sac
- Extending the thoracotomy wound to the posterior axillary line to promote clamshell opening
- 2-handed quality massage
- Aortic occlusion against the spinal column (manual compression)
- Extend the opening of the pericardium as far cranially as possible

6.0 Triage and disposition

- All patients who have undergone successful active resuscitation should be triaged to the most appropriate receiving unit based on patient stability and clinical need. Pre-alert the hospital requesting cardiothoracic support and massive blood loss protocol.
- If despite maximal resuscitation the situation appears hopeless, then life may be pronounced extinct on scene.

7.0 Documentation and audit

- Clear documentation of the procedure should be included in the patient care record and in HEMSbase.
- Cases must be debriefed by the clinical team as soon as possible after the intervention. All thoracotomy cases should be reviewed at a death and disability meeting.

8.0 References

8.1 British Journal of Surgery 2012 Apr; 99 (4) : 541-8. Resuscitative emergency thoracotomy in a Swiss trauma centre. T Lustenberger, L Labler, J F Stover, M J B Keel
PMID: 22139553

8.2 Interactive Cardiovascular and Thoracic Surgery 2013 Apr; 16 (4) : 509-16.
Is there any role for resuscitative emergency department thoracotomy in blunt trauma?
Maziar Khorsandi, Christos Skouras, Rajesh Shah PMID: 23275145

8.3 Annals of Emergency Medicine 2015 Mar; 65 (3) : 297-307.e16.
To be blunt: are we wasting our time? Emergency department thoracotomy following blunt trauma: a systematic review and meta-analysis.David Slessor, Simon Hunter PMID: 25443990

8.4 Thirteen survivors of prehospital thoracotomy for penetrating trauma: a prehospital physician-performed resuscitation procedure that can yield good results. Davies GE, Lockey DJ. J Trauma. 2011 May;70(5):E75-8.

8.5 Pre-hospital thoracotomy: a radical resuscitation intervention come of age? Lockey DJ, Davies G. Resuscitation. 2007 Dec;75(3):394-5.

8.6 Emergency Thoracotomy: “How to do it”. Wise D, Davies G, Coats T, Lockey D, Hyde A, Good A. Emerg Med J. 2005 Jan;22(1):22-4
